# Novel creatine formulation exhibits similar serum creatine concentrations and pharmacokinetics to creatine monohydrate in healthy adults

**DOI:** 10.1080/15502783.2026.2745618

**Published:** 2026-11-09

**Authors:** Gabrielle M. DelBiondo, Celia W. Gottlieb, Paul A. Baker, W. Mikey Johnson, Caroline G. Kelly, Abbie E. Smith-Ryan

**Affiliations:** a Applied Physiology Laboratory, Department of Exercise and Sport Science, University of North Carolina, Chapel Hill, SC, USA; b POWHER Institute, Performance Optimization for Women’s Health and Excellence in Research, Chapel Hill, SC, USA; c Department of Health Science, Human Movement Science Curriculum, University of North Carolina, Chapel Hill, SC, USA; d Department of Nutrition, Gillings School of Public Health, University of North Carolina, Chapel Hill, SC, USA

**Keywords:** Bioavailability, absorption kinetics, supplementation, gastrointestinal tolerability, ergogenic aids, dietary supplements

## Abstract

**Background:**

Creatine monohydrate (CrM) is the most widely studied creatine form, demonstrating robust efficacy, bioavailability, and safety. With creatine growing in popularity, alternative formulations of creatine have been developed to target solubility and to optimize absorption pharmacokinetics (PK), as well as to reduce gastrointestinal (GI) intolerability. The purpose of the current study was to evaluate the acute effects of a novel creatine monohydrate (OptiCreatine^TM^; CrO) compared to CrM on serum creatine PK and GI tolerability in healthy adults.

**Methods:**

Healthy, recreationally active adults (age: 22 ± 3 years; males *n* = 8, females *n* = 9) consumed a single 5-gram dose of CrM or CrO across two separate visits following an overnight fast, in a randomized, double-blind, crossover design. Venous blood samples were collected at baseline and 30, 60, 90, 120, 180, 240, and 300 mins post-ingestion of their assigned treatment to assess serum creatine concentrations. PK parameters including peak concentration (Cmax), time to peak (Tmax), absorption rate constant (k_a_), elimination rate constant (k_el_; h^−1^), and area under the curve (AUC₀–T) were derived using nonlinear regression modeling. GI symptoms were assessed using validated questionnaires.

**Results:**

There was no difference in serum creatine concentrations between CrM or CrO across all participants (*p* = 0.707), males (*p* = 0.318), or females (*p* = 0.996) at any time point. Serum creatine increased over time regardless of treatment (*p* < 0.0001). No differences were observed between CrM and CrO for Cmax (mean difference ± standard deviation (MD ± SD): 7.66 ± 50.37μM; *p* = 0.82), Tmax (MD ± SD: −0.04 ± 0.13 h; *p* = 0.54), k_a_ (MD ± SD: −0.15 ± 0.45 h^−1^; *p* = 0.51), k_el_ (MD ± SD: 0.01 ± 0.03 h^−1^; *p* = 0.61), or AUC₀–T (MD ± SD: 22.09 ± 160.57 μM·h; *p* = 0.74), with no sex × treatment interactions (p ≥ 0.05). Descriptive evaluation showed similar GI symptom profiles between conditions, with low frequency and minimal severity reported across both treatments.

**Conclusions:**

A single 5-gram dose of CrO produced similar serum creatine and PK characteristics to CrM in healthy adults. These findings support CrO as a viable delivery option, with no significant differences in acute pharmacokinetic profiles or GI tolerability compared to CrM.

## 1. Introduction

Creatine supplementation is well documented for its host of performance benefits (i.e. strength, power, and lean mass) [1,2], and emerging health benefits including improved cognitive function and enhanced blood flow [3,4]. Among available forms, creatine monohydrate (CrM) is the most extensively studied and widely used due to its proven bioavailability, efficacy, and high safety profile [5,6]. Despite the established absorption efficiency of CrM, interest in alternative creatine formulations has been driven by factors such as solubility and dispersion characteristics, and the potential to modify absorption kinetics in circulating creatine responses [7].

Pharmacokinetics (PK), defined as the time course of nutrient absorption, distribution, and elimination, provides a quantitative framework to characterize circulating creatine responses, including peak concentration, time to peak, and overall exposure, which can be modeled to describe absorption and clearance dynamics [8,9]. Previous research has demonstrated that CrM continues to have the highest bioavailability amongst creatine types, with variability across different types of creatine [10]. One previous study demonstrated a greater higher mean peak concentration after ingestion of creatine pyruvate [mean ± standard deviation (SD): 968 ± 82.3 mM] compared to both creatine citrate (837 ± 71.5 mM) and CrM (751 ± 32.9 mM) [11], demonstrating pharmacokinetic differences. With the increasing applications and demand for creatine supplementation, alongside new technology, new forms and delivery mechanisms of creatine are being developed. Due to the high bioavailability of CrM, the most compelling justification for novel creatine forms is improved physicochemical characteristics such as aqueous solubility, which may enhance mixability, palatability, and delivery versatility without compromising exposure. Improved solubility could reduce precipitation/sedimentation in beverages and enable ready-to-drink or lower-volume dosing formats that may improve adherence in both athletic and clinical settings [5,11]. From a PK perspective, more rapid dissolution may also reduce variability in time-to-peak (Tmax) and early concentration profiles, helping to better characterize absorption kinetics while maintaining comparable overall exposure (AUC) to CrM [8,9].

Novel creatine formulations with altered physicochemical properties, such as OptiCreatine^TM^ (CrO), continue to emerge. Despite increased consumer use and product innovation, there is limited scientific evidence evaluating changes in absorption kinetics, systemic exposure, or GI tolerability. Rigorous acute PK comparisons are required to establish bioequivalence and to quantify any differences in the concentration–time profile and overall exposure that may be significant for dosing and real-world adherence. To address this scientific gap, the present randomized, double-blind, cross-over study compared a novel creatine formulation (CrO) compared to CrM on acute serum creatine concentrations and PK parameters (Cmax, AUC, Tmax) in healthy, recreationally active males and females.

## 2. Materials and methods

### 2.1. Participants

Seventeen recreationally active males (*n* = 8) and females (*n* = 9) were enrolled in this study (Table 1; Figure 1). Fitness status was determined via a comprehensive health history questionnaire, including the International Physical Activity Questionnaire (IPAQ-short, [12]). Participants were eligible if they were between 18 and 45 years of age and had a body mass index (BMI) < 35 kg·m^−2^. Exclusion criteria included a diagnosis of chronic kidney, liver, or obstructive pulmonary disease, cancer, or current use of medications known to influence fluid balance or metabolism (e.g. diuretics, corticosteroids), as well as any type of creatine supplementation within two months prior to enrollment. All participants provided written informed consent prior to participation. The study was approved by the University Institutional Review Board (IRB #25-2072) and conducted in accordance with the Declaration of Helsinki. The study was registered at Clinicaltrials.gov (NCT07225335).

**Figure 1.**
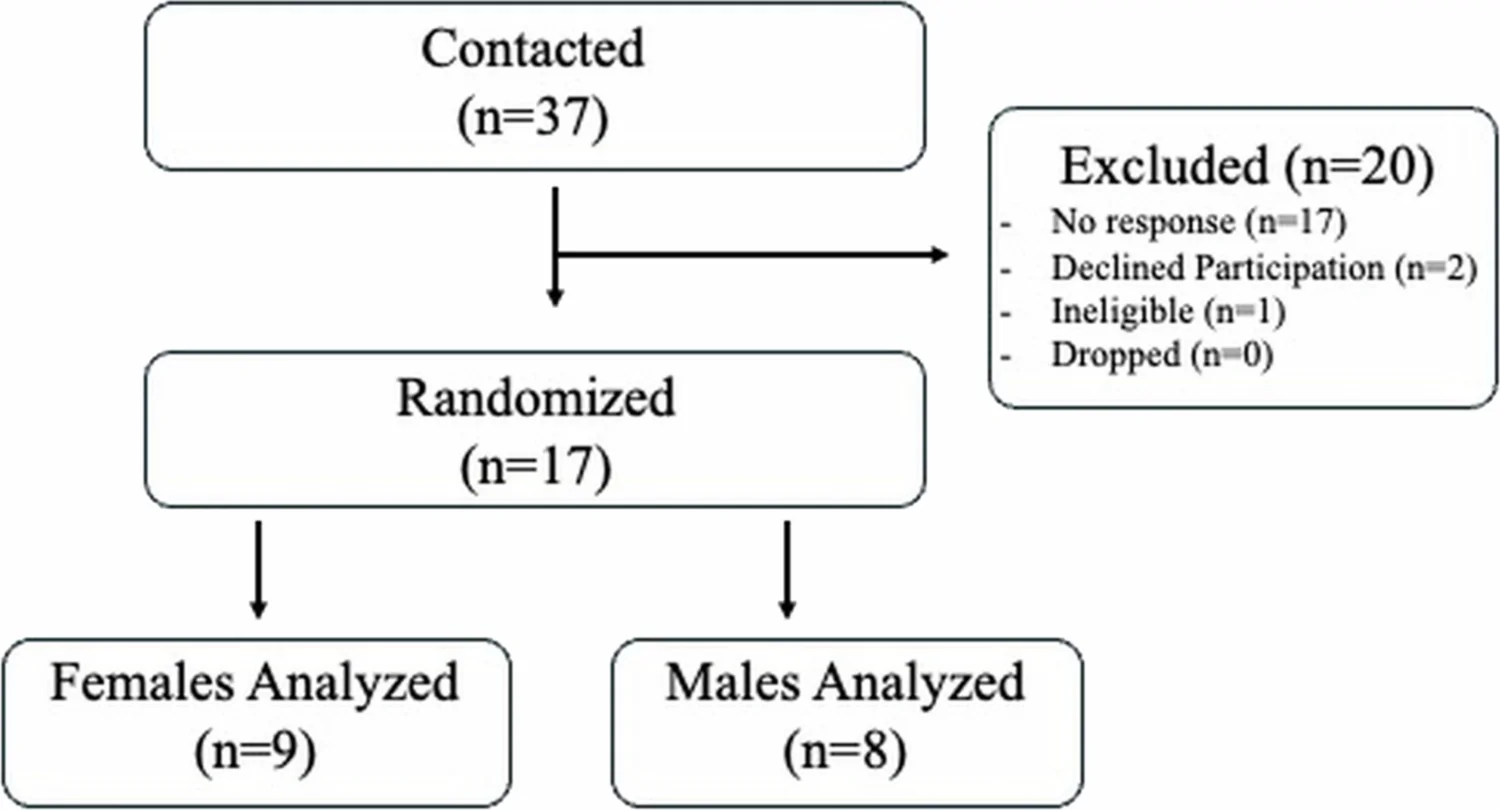
CONSORT diagram.

**Table 1.** Demographics of the total sample.

Participants	Age (yrs)	Height (cm)	Weight (kg)	BMI (kg/m^2^)
All (*n* = 17)	22.88 ± 2.78	170.06 ± 7.29	70.79 ± 9.56	24.41 ± 2.35
Male (*n* = 8)	21.63 ± 2.50	176.46 ± 4.14	78.81 ± 6.88	25.31 ± 2.32
Female (*n* = 9)	24.00 ± 2.65	164.37 ± 3.74	63.66 ± 4.46	23.61 ± 2.19

Values are presented as mean ± standard deviation. Seventeen recreationally active individuals (n = 17; males n = 8, females n = 9) between 18-45 years of age were included in the study. Body mass index (BMI) was calculated as kg·m^−2^.

### 2.2. Experimental design

This study utilized a randomized, double-blind, crossover design (Figure 2). Participants completed two acute testing visits separated by a minimum 72-hour washout period. For each visit, participants arrived at the laboratory following an 8-hour fast and abstention from caffeine and alcohol for 12 hours and strenuous exercise for 24 hours. Upon arrival, participants provided a urine sample for assessment of hydration status via urine specific gravity (refractometer; a.u.). Anthropometric measurements were obtained using a stadiometer (Perspective Enterprises, Portage, MI, USA).

**Figure 2.**
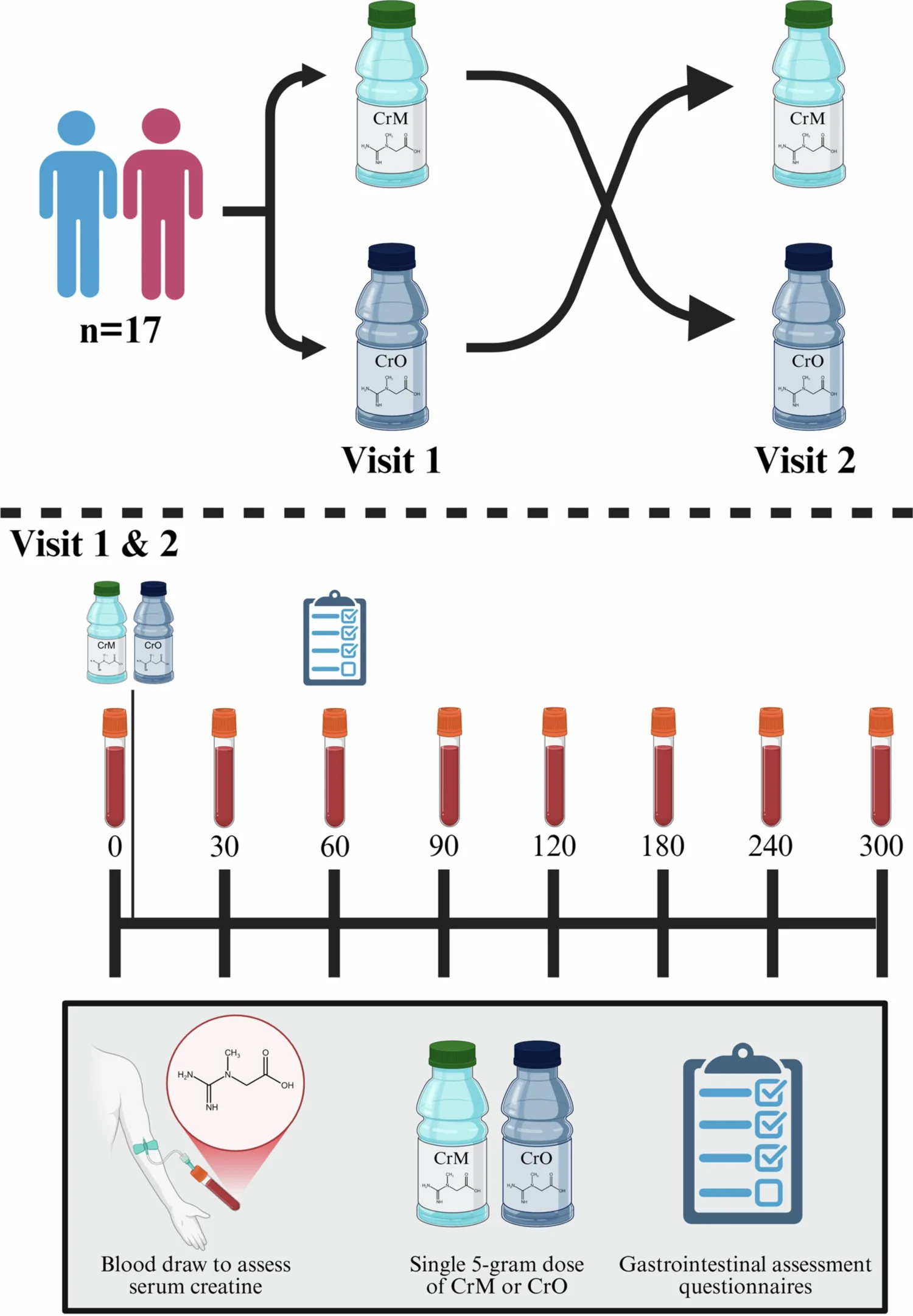
Experimental design.

### 2.3. Supplementation

Participants were randomized to treatment order using computer-generated allocation software (Sealedenvelope™, London, UK) with stratification by sex. All participants completed both supplement conditions in a crossover manner. During each acute visit, participants consumed a single 5-gram dose of either CrM or 5-gram dose of CrO (OptiCreatine^TM^; TSI Group LTD, Ames, Iowa, USA) mixed with 4–6 oz of water immediately following the baseline blood draw and consumed within 10 minutes of being dissolved. CrM was obtained from a commercial supplier (Shandong Changrui Biotech Co., Ltd., Shandong, China) and verified via certificate of analysis (COA) for purity (99.75%) and acceptable limits of creatinine, dicyandiamide, and heavy metals prior to use. OptiCreatine™ (CrO) was provided by the manufacturer (TSI Group Ltd., Ames, IA, USA), with COA resulting in 100.4% purity. Treatments were mixed in opaque containers, by a team member not involved in participant interaction or data collection. Both participants and investigators were blinded to treatment allocation.

### 2.4. Serum creatine concentrations

Serum creatine concentrations were determined from blood collect via intravenous catheter sampling from an antecubital vein, placed with participants in a supine position. A baseline blood sample (5 mL) was collected immediately following catheter placement (baseline; 0 min). Subsequent blood samples were obtained at 30, 60, 90, 120, 180, 240, and 300 minutes following supplement ingestion. Prior to each collection, a 3 mL waste sample was drawn and discarded, followed by sample collection and a saline flush (10 mL). Whole blood samples were allowed to clot at room temperature for 50 minutes before centrifugation at 2500 rpm for 20 minutes at 23 °C. Serum was aliquoted and stored at −80 °C until analysis. Samples were analyzed by LabCorp (Burlington, NC) using a clinically validated assay, with reported assay precision (coefficient of variation) of approximately 10–15%.

### 2.5. Pharmacokinetic analysis

Pharmacokinetic analysis modeling parameters were estimated using a one-compartment oral absorption model with first-order absorption and elimination (Bateman function), consistent with previously established approaches [11,13]. Model fitting was performed on creatine concentrations above baseline (C − C₀), with non-positive values excluded to improve stability of nonlinear parameter estimation. Nonlinear regression was conducted using a Levenberg–Marquardt algorithm to derive peak concentration (Cmax; μM above baseline), time to peak (Tmax; h), absorption rate constant (k_a_; h^−1^), elimination rate constant (k_el_; h^−1^), total area under the curve from 0 to the last observed time point (AUC₀–T; μM·h).

### 2.6. Gastrointestinal assessments

Gastrointestinal symptoms were assessed 60 minutes post-ingestion using validated questionnaires as an exploratory analysis, including the Digestion-Associated Quality of Life Questionnaire (DQLQ), Gastrointestinal Symptom Rating Scale (GSRS), and a validated bloating questionnaire. Assessments were completed electronically during each acute visit following the 5-gram dose.

### 2.7. Statistical analysis

Serum creatine concentrations were converted from mg·dL^−1^ to μM using the molecular weight of creatine (131.13 g·mol^−1^), consistent with established methods [14]. Sampling times were expressed relative to ingestion and standardized to 0, 30, 60, 90, 120, 180, 240, and 300 min to represent the 0–5 h post-ingestion period. The serum creatine concentration–time response was analyzed using a two-way repeated-measures analysis of variance (RM-ANOVA) with within-subject factors of Treatment (CrM, CrO) and Time (8 levels), including the treatment × time interaction. When significant main or interaction effects were detected, pairwise comparisons were performed with Bonferroni-adjusted post hoc tests.

Pharmacokinetic outcomes were a derived within each participant and condition using using a one-compartment oral absorption model with first-order absorption and elimination (Bateman function), consistent with previously established approaches [11,13]. Total systemic exposure over 0–5 h (AUC0–5h) was calculated using the trapezoidal method from 0 to 300 min. Between-condition differences in PK parameters (Cmax, Tmax, AUC0–5h) were derived using nonlinear regression modeling. A sample size of *n* = 16 was determined a priori with creatine serum concentration between CrM and CrO as the primary outcome to achieve a power of 0.8, nonsphericity correction of 1, and a significance level of 0.05. Statistical significance was set a priori at *α* = 0.05 (two-tailed). Analyzes were performed using IBM SPSS Statistics (IBM Corp., Armonk, NY, USA; version 29.0).

## 3. Results

### 3.1. Serum creatine

There was no difference in serum creatine concentration between CrM and CrO at any timepoint between all participants (time × treatment interaction, *p* = 0.707; Figure 3A), males (time × treatment interaction, *p* = 0.318; Figure 3B), or females (time × treatment interaction, *p* = 0.996; Figure 3C). Regardless of treatment there was an increase in serum creatine concentrations over time for all participants (time, *p* < 0.0001), excluding time 0. There were no differences in changes in serum creatine concentrations from baseline (Δmg/dL) in all participants: 30 min (CrM: 5.62 ± 2.57 mg/dL; CrO: 5.85 ± 4.19 mg/dL), 60 min (CrM: 12.48 ± 2.22 mg/dL; CrO: 11.15 ± 3.41 mg/dL), 90 min (CrM: 11.68 ± 1.68 mg/dL; CrO: 11.25 ± 2.35 mg/dL), 120 min (CrM: 9.51 ± 1.90 mg/dL; CrO: 9.88 ± 2.33 mg/dL), 180 min (CrM: 5.89 ± 1.74 mg/dL; CrO: 5.85 ± 1.77 mg/dL), 240 min (CrM: 3.85 ± 1.75 mg/dL; CrO: 3.95 ± 1.73 mg/dL), or 300 min (CrM: 2.65 ± 1.40 mg/dL; CrO: 2.67 ± 1.28 mg/dL; time × treatment interaction, *p* = 0.711; Table 2). No differences in changes from baseline in serum creatine concentrations were observed in males (time × treatment interaction, *p* = 0.319) or females (time × treatment interaction, *p* = 0.989) (Table 2).

**Figure 3.**
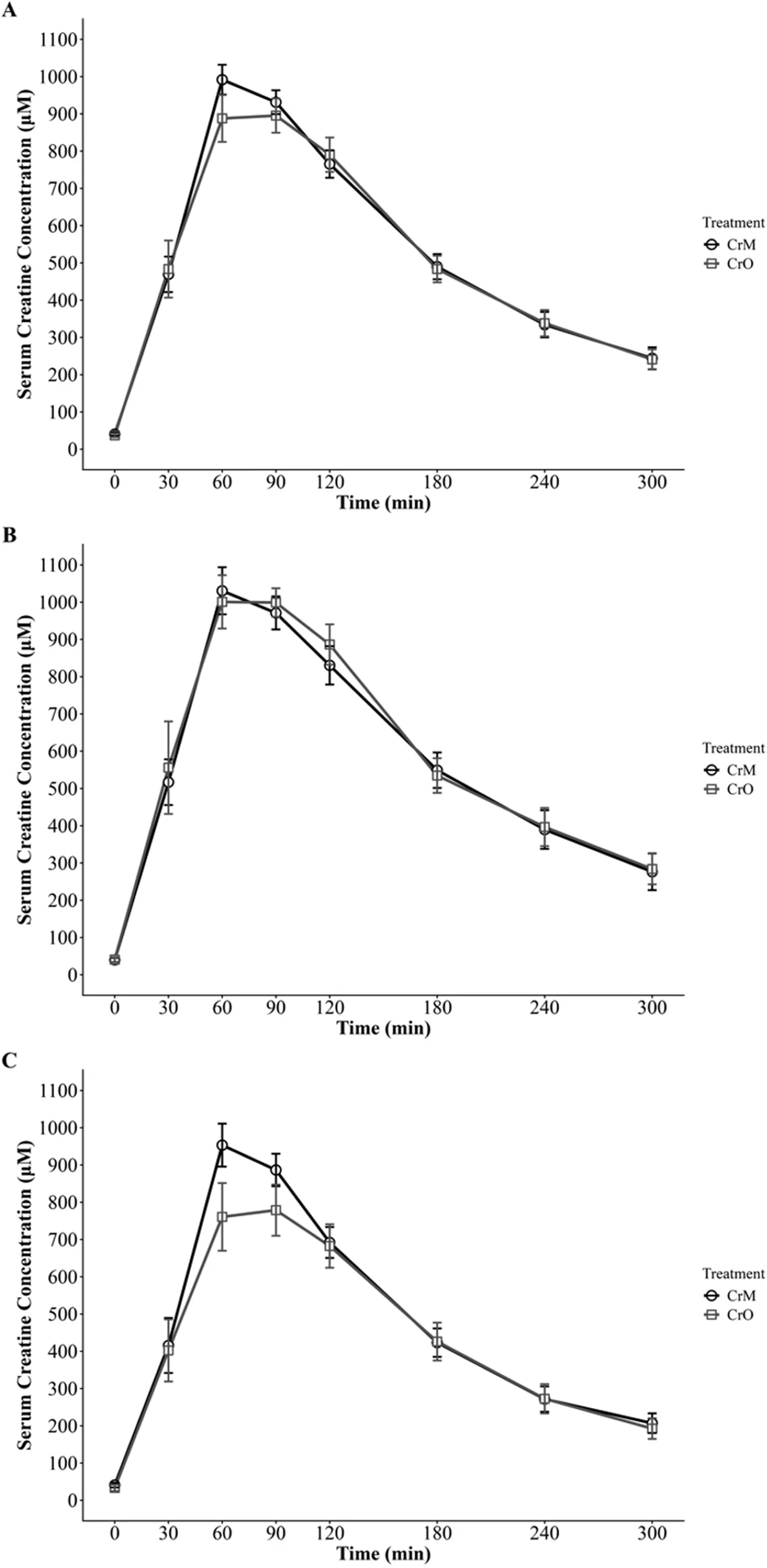
Serum creatine concentration responses following ingestion of creatine monohydrate (CrM) and a novel creatine (CrO). Serum creatine concentrations (μM) are shown across a 0–300 min post-ingestion period for (**A**) all participants (*n* = 17), (**B**) females (*n* = 9), and **(C)** males (*n* = 8). Values are presented as mean ± standard error. Participants completed both conditions in a randomized, double-blind, crossover design. No time × treatment interactions were observed for all participants (*p* = 0.707), males (*p* = 0.318), or females (*p* = 0.996). A significant main effect of time was observed in all groups (*p* < 0.0001).

**Table 2.** CrM and CrO timepoint analysis as mean difference in blood serum creatine from baseline.

	All		Males		Females	
Time (min)	CrM (*n* = 17)	CrO (*n* = 17)	CrM (*n* =8)	CrO (*n* = 8)	CrM (*n* = 9)	CrO (*n* = 9)
30	5.62 ± 2.57	5.85 ± 4.19	4.91 ± 2.79	4.84 ± 3.15	5.78 ± 2.66	6.76 ± 4.94
60	12.48 ± 2.22	11.15 ± 3.41	11.96 ± 2.11	9.54 ± 3.40	12.80 ± 2.27	12.59 ± 2.87
90	11.68 ± 1.68	11.25 ± 2.35	11.09 ± 1.48	9.78 ± 2.34	12.22 ± 1.64	12.57 ± 1.47
120	9.51 ± 1.90	9.88 ± 2.33	8.54 ± 1.35	8.51 ± 1.93	10.26 ± 1.88	11.09 ± 2.01
180	5.89 ± 1.74	5.85 ± 1.77	5.01 ± 1.31	5.15 ± 1.70	6.53 ± 1.72	6.48 ± 1.68
240	3.85 ± 1.75	3.95 ± 1.73	3.03 ± 1.11	3.14 ± 1.24	4.45 ± 1.88	4.67 ± 1.85
300	2.65 ± 1.40	2.67 ± 1.28	2.16 ± 0.75	2.09 ± 0.85	3.033 ± 1.62	3.19 ± 1.42

Values represent change from baseline (0 min), expressed as mean ± SD. All concentrations are presented in mg/dL. For all participants, males, and females time *p* < 0.0001. There is no significance for any treatment or time × treatment interaction (*p* ≥ 0.05).

#### 3.1.1. Pharmacokinetics

No differences were observed for Cmax [mean difference (MD) ± SD: CrM-CrO: 7.66 ± 135.63 μM; 95% Confidence Interval (CI): −77.39, 62.08, *p* = 0.82], Tmax (MD ± SD: −0.04 ± 0.29 hr; 95% CI: −0.10, 0.19; *p* = 0.54), k_a_ (MD ± SD: −0.15 ± 0.90 h^−1^; 95% CI: −0.32, 0.61; *p* = 0.51), k_el_ (MD ± SD: 0.01 ± 0.71 h^−1^; 95% CI: −0.05, 0.03; *p* = 0.61), or AUC₀-T (MD ± SD: 22.09 ± 272.38 μM·h; 95% CI: −162.14, 117.95; *p* = 0.74) (Table 3). No sex × treatment interactions were observed for between-condition in pharmacokinetics (*p* ≥ 0.05).

**Table 3.** Pharmacokinetic parameters derived from serum creatine concentrations following ingestion of CrM and CrO.

	CrM	CrO	Mean difference	*P*-value
	(*n* = 17)	(*n* = 17)		t-test
Cmax (uM)	742.41 ± 105.64	734.75 ± 178.80	7.66 ± 50.37	0.82
Tmax (h)	1.14 ± 0.321	1.19 ± 0.434	−0.04 ± 0.131	0.54
k_a_ (h^−1^)	2.29 ± 0.910	2.43 ± 1.597	−0.15 ± 0.446	0.51
k_el_ (h^−1^)	0.28 ± 0.0947	0.27 ± 0.0684	0.01 ± 0.0283	0.61
AUC₀–T (μM·h)	2513.67 ± 404.14	2491.58 ± 524.40	22.09 ± 160.57	0.74

Values are presented as mean ± standard deviation. Participants (n = 17) completed both conditions in a randomized, double-blind, crossover design. Serum creatine concentrations were used to derive pharmacokinetic parameters using a one-compartment model with first-order absorption and elimination, including maximal concentration (Cmax; μM above baseline), time to maximal concentration (Tmax; h), absorption rate constant (k_a_; h⁻¹), elimination rate constant (k_el_; h⁻¹), and area under the curve from time 0 to the final observed timepoint (AUC₀–T; μM·h). Mean differences are presented as CrM minus CrO. *P*-values reflect paired comparisons between conditions.

#### 3.1.2. Gastrointestinal assessments

Descriptive gastrointestinal symptom response patterns for CrO and CrM are presented in Table 4. GI symptoms were generally minimal across both treatments, with low overall burden and no differences in bowel habits. CrM was associated with slightly more frequent reports of gas/flautus and rumpling. CrO showed a higher occurrence of mild nausea. Bloating was reported under both formulas.

**Table 4.** Gastrointestinal symptom profile and exploratory tolerability descriptive responses comparing CrM and CrO based on GSRS and DQLQ outcomes.

Symptom domain		GSRS question (Item)	Observed response pattern
Gas/Flatus	“Have you been bothered by PASSING GAS OR FLATUS during the past week?”	CrM columns show more frequent non-zero responses and slightly higher categorical ratings (e.g. minor → mild); CrO responses cluster at none/minor	CrM > CrO
Rumbling/GI Movement	“Have you been bothered by RUMBLING in your stomach during the past week?”	Sparse or minimal endorsements in CrO; more frequent endorsements in CrM, temporally aligned with acute dosing	CrM > CrO
Bloating	“Has your stomach felt BLOATED during the past week?”	Bloating reported under both treatments; CrO bloating often coincides with menstrual timing; CrM bloating more often co-occurs with elevated gas/rumbling in same rows	Shared signal; contextual difference
Nausea	“Have you been bothered by NAUSEA during the past week?”	Non-zero nausea responses appear more frequently in CrO; CrM nausea is less frequent and lower in magnitude	CrO > CrM
Bowel Habits	GSRS constipation, diarrhea, stool consistency items	Responses remain minimal and stable across treatments and phases	No difference
Functional Impact	DQLQ activity, eating, social items	Responses largely “never” or “rarely” across all conditions	No difference

Symptom domains are derived from the Gastrointestinal Symptom Rating Scale (GSRS) and Digestive Quality of Life Questionnaire (DQLQ). Data are presented descriptively as observed response patterns across conditions, with treatment signals indicating the relative frequency or magnitude of reported symptoms between formulations. No statistical comparisons were performed.

## 4. Discussion

The current study examined the acute serum creatine concentrations and pharmacokinetics of a novel creatine formulation (CrO) compared to CrM in normal healthy males and females. The major findings of the present study demonstrate that across a 0–300 minute window (**1**) serum creatine concentrations rose over time but did not differ between CrM and CrO at any time point and (**2**) there was no difference in PK parameters between CrM and CrO; Taken together, these findings indicate that a 5-gram dose of CrM and CrO exhibit similar acute bioavailability, PK exposure, and tolerability, in healthy males and females.

### 4.1. Serum creatine response

The present findings indicate that the novel CrO creatine formulation explored in the present study provides a comparable alternative to CrM, achieving similar serum creatine levels after a single 5-gram dose. Comparative data on the absorption of alternative creatine formulations relative to CrM are limited, with approximately 88% of commercially available creatine products lacking sufficient evidence to support claims of enhanced bioavailability, efficacy, or safety [15]. Only three studies were found to have examined alternative creatine forms compared to CrM, making efficacy comparisons difficult [7]. The three available studies compared magnesium creatine chelate [16], creatine ethyl ester [17], and creatine pyruvate [11] in comparison to CrM and all reported no significant differences on absorption, or tolerability, similar to the present study with CrO.

In the current study, peak serum creatine concentrations occurred at 60-90 minutes following supplementation for both treatments, which closely mirrors previous data showing peaks at approximately 1.9 hours [13] and 2 hours [18], respectively. Both formulations in the current study mapped closely to the 0–5 hour concentration profile reported in the literature, with peak concentrations observed within ~120 minutes post-ingestion followed by a decline towards baseline, reinforcing practical comparability [11,18]. While some studies have reported statistically significant differences, such as creatine pyruvate producing 17% higher peak concentrations and 14% greater area under the curve compared to CrM, the authors concluded that these small kinetic differences are unlikely to meaningfully influence muscle creatine accumulation during loading [11].

### 4.2. Pharmacokinetics

PK parameters (Cmax, Tmax, AUC, elimination rate) did not differ between CrM and CrO, supporting pharmacokinetic equivalence in absorption, distribution, and systemic exposure in the current study. Creatine PK are nonlinear and influenced by skeletal muscle saturation status, with clearance decreasing at steady-state due to finite muscle storage capacity [13]. As such, even if small kinetic differences were present, their impact on muscle creatine accretion or performance outcomes would likely be minimal [19]. The present data suggest that modifications to creatine formulation do not meaningfully alter acute serum concentration-time profile compared to CrM, and seem to be comparable. These findings align with prior work demonstrating that alternative forms of creatine do not confer meaningful advantages over CrM in terms of absorption, tolerability, or performance [6]. The absence of significant differences suggests that novel formulations, including CrO performs comparably to CrM without compromising PK outcomes, which may be beneficial for future form factors of creatine usage.

### 4.3. Gastrointestinal assessments

Descriptive evaluation of GI symptom responses revealed modest observations in symptom patterns between CrO and CrM. Reports of gas/flatulence and rumbling were more frequently endorsed 60 min following CrM (*n* = 7) compared to CrO (*n* = 6) which was characterized by a higher frequency of non-zero responses and clustering within minor severity categories. Bloating was reported under both conditions with similar frequency (*n* = 4). In contrast, nausea responses were more frequently reported with CrO (*n* = 4) compared to CrM (*n* = 3); however, these were generally low in magnitude and transient. Across both treatments, bowel habit measures (constipation, diarrhea, stool consistency) remained minimal and stable, and functional impact on daily activities, eating behavior, and social engagement was largely reported as “never” or “rarely,” indicating no meaningful disruption to daily function. These findings are consistent with the established safety profile of CrM [6]. Even with long-term supplementation (average 310 days at 10 g/day) of CrM, no differences in adverse gastrointestinal events with CrM compared to placebo were reported, including nausea (23% vs 24%), gastrointestinal discomfort (19% vs 18%), and diarrhea (35% vs 24%) [20]. The few GI disturbances that are reported in healthy individuals commonly occur with higher intake levels (e.g. 20 g/day) [21,22]. Taken together, the transient and low-magnitude symptom profiles observed, along with the absence of functional impairment, indicate that single 5-gram doses are well tolerated under acute dosing conditions.

### 4.4. Limitations

Notable limitations of the current study relate to the acute nature of this study (0–300 minutes post-ingestion) limits interpretation to immediate PK responses and does not reflect longer-term absorption with chronic creatine supplementation. Further, future research should examine chronic supplementation to determine whether similar acute absorption and PK translate to comparable effects on muscle creatine content, performance, and health outcomes across diverse populations.

## 5. Conclusions

In conclusion, the present study demonstrates that a single 5-gram dose of both CrM and the novel CrO formulation produce similar acute time-course serum creatine responses and PK outcomes in healthy, recreationally active adults. No differences were observed in peak concentration, time to peak, or overall systemic exposure (absorption and elimination rates), indicating highly-similarity in acute bioavailability between CrM and CrO. These findings suggest that differences in formulation can be explored without necessarily compromising systemic creatine exposure, supporting continued investigation into next-generation creatine formulations that may improve mixing characteristics, product versatility, and user adherence. Future work should extend these observations to repeated dosing and longer-term supplementation, with attention to GI tolerability.

## Data Availability

Deidentified individual data that supports the results will be shared beginning 24 months following publication provided the investigator who proposes to use the data has approval from an Institutional Review Board (IRB), Independent Ethics Committee (IEC), or Research Ethics Board (REB), as applicable, and executes a data use/sharing agreement with UNC. When data sharing is permitted, a Data Use Agreement (DUA) specifying the uses of such data to be shared must be in place before any data is shared. Requests can be submitted to the Industry Contracting for processing.
